# Spike‐and‐wave discharges of absence seizures in a sleep waves‐constrained corticothalamic model

**DOI:** 10.1111/cns.14204

**Published:** 2023-04-10

**Authors:** Martynas Dervinis, Vincenzo Crunelli

**Affiliations:** ^1^ Neuroscience Division, School of Bioscience Cardiff University Museum Avenue Cardiff CF10 3AX UK; ^2^ Present address: School of Physiology, Pharmacology and Neuroscience Biomedical Building Bristol BS8 1TD UK

**Keywords:** cortex, GABA‐A inhibition, GABA‐B inhibition, thalamus, T‐type Ca^2+^ channels

## Abstract

**Aims:**

Recurrent network activity in corticothalamic circuits generates physiological and pathological EEG waves. Many computer models have simulated spike‐and‐wave discharges (SWDs), the EEG hallmark of absence seizures (ASs). However, these models either provided detailed simulated activity only in a selected territory (i.e., cortical or thalamic) or did not test whether their corticothalamic networks could reproduce the physiological activities that are generated by these circuits.

**Methods:**

Using a biophysical large‐scale corticothalamic model that reproduces the full extent of EEG sleep waves, including sleep spindles, delta, and slow (<1 Hz) waves, here we investigated how single abnormalities in voltage‐ or transmitter‐gated channels in the neocortex or thalamus led to SWDs.

**Results:**

We found that a selective increase in the tonic γ‐aminobutyric acid type A receptor (GABA‐A) inhibition of first‐order thalamocortical (TC) neurons or a selective decrease in cortical phasic GABA‐A inhibition is sufficient to generate ~4 Hz SWDs (as in humans) that invariably start in neocortical territories. Decreasing the leak conductance of higher‐order TC neurons leads to ~7 Hz SWDs (as in rodent models) while maintaining sleep spindles at 7–14 Hz.

**Conclusion:**

By challenging key features of current mechanistic views, this simulated ictal corticothalamic activity provides novel understanding of ASs and makes key testable predictions.

## Graphical abstract

Using a biophysical large‐scale corticothalamic model that reproduces the EEG sleep waves, including sleep spindles, delta, and slow (<0.1 Hz) waves, we investigated how single abnormalities in voltage‐ or transmitter‐gated channels in the neocortex or thalamus lead to spike‐and‐wave discharges (SWDs) of absence seizures in the EEG. We found that a selective increase in the tonic GABA‐A inhibition of first‐order thalamocortical (TC) neurons or a selective decrease in cortical phasic GABA‐A inhibition is sufficient to generate ~4 Hz SWDs (as in humans) that invariably start in neocortical territories. Moreover, decreasing the leak conductance of higher‐order TC neurons leads to ~7 Hz SWDs (as in rodent models) while maintaining sleep spindles at 7–14 Hz.

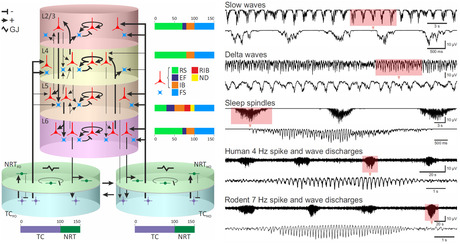

## 1 INTRODUCTION

Absence seizures (ASs) are genetic, generalized, non‐convulsive seizures characterized by sudden, relatively brief lapses of consciousness that are invariably accompanied by spike‐and‐waves discharges (SWDs) in the EEG.^1^, ^2^, ^3^, ^4^, ^5^ It is well established that both the clinical and the electrographic symptoms of ASs originate from aberrant activity of corticothalamic networks^5^ and a number of genetic abnormalities have been identified in humans with ASs.^6^ However, our current understanding of how these genetic deficits lead to the ictal EEG activity observed during ASs is still not fully understood.

Many biophysical and non‐biophysical models have simulated the generation of SWDs leading to increased knowledge of their underlying mechanisms.^7^, ^8^, ^9^, ^10^, ^11^, ^12^, ^13^, ^14^, ^15^, ^16^, ^17^, ^18^, ^19^, ^20^, ^21^ However, these models either provided detailed simulated activity only in a selected territory (i.e., cortical or thalamic) or did not test whether their corticothalamic networks could reproduce physiological activities that are known to be generated by these circuits.^22^ Here, we used our corticothalamic model (Figure S1) that faithfully simulates EEG waves of natural sleep, that is, sleep spindles, delta, and slow (<1 Hz) waves^23^ (Figure S2), to investigate whether single abnormal voltage‐ or transmitter‐gated conductances bring about SWDs of ASs. In particular, we show that an increase in the tonic GABA‐A inhibition of first‐order thalamocortical (TC_FO_) neurons, a decrease in cortical phasic GABA‐A inhibition, an increase in cortical AMPA receptor function, or an increase in the T‐type Ca^2+^ conductance of higher‐order thalamocortical (TC_HO_) neurons generates ~4 Hz SWDs (as observed in humans with ASs^2^, ^5^) that invariably start in the neocortex.

## 2 METHODS

### 2.1 Corticothalamic network model

We used our biophysical model of the corticothalamic network (Figure S1) that faithfully reproduces the typical EEG waves of natural sleep (Figure S2).^23^ Briefly, our corticothalamic model contains 900 model neurons and is organized into six sectors (Figure S1): four cortical layers, including layers 2/3 (L2/3), 4 (L4), 5 (L5), and 6 (L6), and a first‐ and a higher‐order thalamic nucleus with their thalamocortical neurons (TC_FO_ and TC_HO_, respectively) which are reciprocally connected to inhibitory NRT neurons (NRT_FO_ and NRT_HO_, respectively).^24^ Each cortical layer is divided into two subsectors, each with 100 excitatory and 50 inhibitory neurons. Cortical excitatory subsectors contain different numbers of regular spiking (RS), intrinsically bursting (IB), early firing (EF), repetitive intrinsically bursting (RIB), and network driver (ND) neurons, whereas inhibitory subsectors have FS interneurons (Figure S1). The full model is a two‐dimensional stack of subsector neuron rows. The neuron position within a subsector was determined pseudo‐randomly.^23^

### 2.2 Model network connectivity

Connections were organized topographically with sources and targets located in matching regions of their corresponding structures. A neuron did not synapse onto itself and could only form a single synapse on its target neuron. The number of contacts that a source neuron could form in a target structure was defined by the parameter P (a projection radius). Other key connectivity parameters (e.g., connection weight, postsynaptic potential shape, synaptic transmission latency, and synaptic receptors) are described in detail in Dervinis and Crunelli.^23^ Similarly, the numerical values of the various intrinsic and synaptic conductances of the different neuronal populations are detailed in Dervinis and Crunelli.^23^

### 2.3 Simulations

All simulations were carried out in NEURON on a desktop computer or one of the following computing clusters: the Neuroscience Gateway (NSG) Portal for Computational Neuroscience or the Cardiff University School of Biosciences Biocomputing Hub HPC/Cloud infrastructure.

### 2.4 Data analyses

Simulation data were analyzed and visualized with the help of custom‐written Matlab (MathWorks Inc.) routines. The raw EEG signal was filtered and cross‐correlated as described in Dervinis and Crunelli.^23^ SWD Hilbert transform phase was calculated by bandpass filtering raw EEG traces using Butterworth filter with the following parameters: passband and stopband frequencies centered at ±2 Hz and ±4 Hz around the SWD oscillation frequency (~4 or ~7 Hz), respectively, and passband ripple and stopband attenuation being 0.5 and 65 dB, respectively. The filtered EEG signal was then subjected to Matlab's hilbert function. Hilbert phase synchronization index (PSI)^25^ was calculated for two filtered signals obtained using the same filtering parameters as above and then smoothed using a moving average window of 1 s duration (for additional data analyses, see Appendix S1 in Dervinis and Crunelli^23^).

## 3 RESULTS

As shown in the preceding paper,^23^ our thalamocortical model is capable of smoothly transitioning between wakefulness (as evident from a low‐amplitude, high‐frequency EEG) and different EEG waves of natural sleep (depending on the input resistance of its constituent neurons) and it does not enter an overly synchronous activity‐mode typical of seizures. However, one particular state of the model is prone to generate ictal states, that is, the transition between sleep and wakefulness. When the model is in this state, different single‐membrane conductance changes in either cortical or thalamic neurons do lead to an EEG waveform typical of SWDs of ASs, as described below. Notably, all the changes in different conductances that lead to simulated SWDs have a minimal impact on sleep waves (not shown).

### 3.1 Selective increase in tonic GABA‐A inhibition of TC_FO_ neurons generates SWDs

**FIGURE 1:**
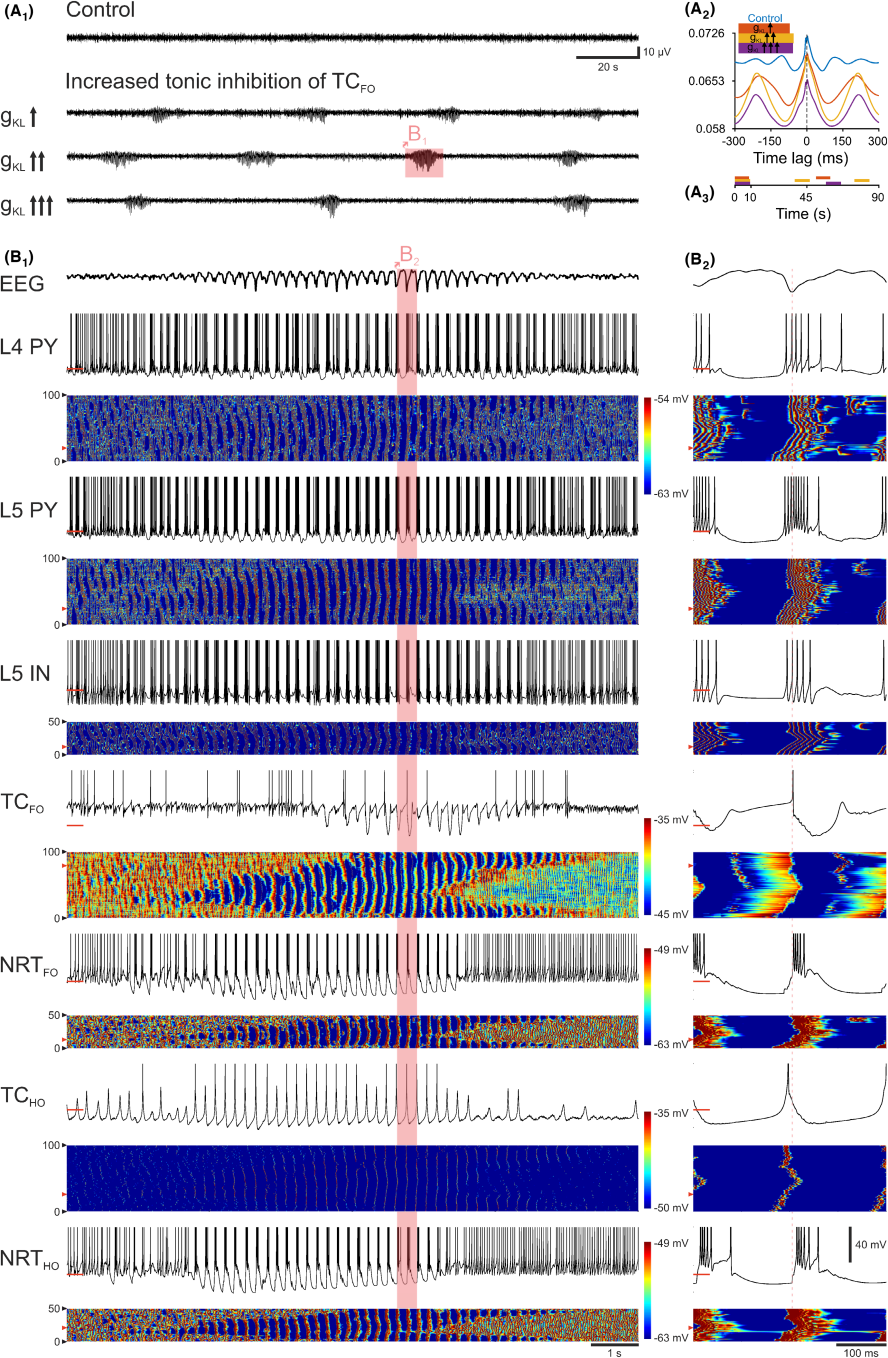
Selective increase in tonic GABA‐A inhibition of TC_FO_ neurons elicits SWDs. (A_1_) EEG traces show the induction of spontaneous SWDs at ~4 Hz after progressive increases in g_KL_ of TC_FO_ neurons, mimicking the constitutively high tonic GABA‐A inhibition reported in AS models. The control condition shows simulated desynchronized state typical of relaxed wakefulness. The SWD in the shaded area is expanded in B_1_. (A_2_) Cross‐correlations between APs of all neurons and the EEG calculated over a 20 min simulation period. Shaded regions represent 95% confidence intervals. (A_3_) Schematic timeline showing ictal and interictal periods for different g_KL_ values. Color code as in (A_2_). (B_1_) Top trace: EEG. Panels below show the membrane potential (upper trace) of the indicated neuron and a color‐coded graph of the membrane potential of all neurons of the indicated population. Red bars on the membrane potential traces indicate −60 mV. Red arrowheads in the color‐coded graphs mark the neuron shown in the corresponding membrane potential trace. (B_2_) Same as (B_1_), shows the expanded SWD cycle highlighted in (B1). Vertical red dotted line marks the peak of the SWD spike. L4 PY, Pyramidal neuron in cortical layer 4; L5 PY, pyramidal neuron in cortical layer 5; L5 IN, interneuron in cortical layer 5; TC_FO_, first‐order TC neuron; TC_HO_, higher‐order TC neuron; NRT_FO_, first‐order NRT neuron; NRT_HO_, higher‐order NRT neuron.

Evidence in mouse and rat AS models have shown that an increased tonic GABA‐A inhibition of TC_FO_ neurons (that results from higher thalamic GABA levels^26^) is necessary and sufficient for AS generation.^27^, ^28^, ^29^ Moreover, higher levels of GABA were found in the thalamus of a child with ASs,^30^ and drugs that are known to increase GABA levels, that is, vigabatrin and tiagabine, can induce or aggravate ASs in humans.^31^, ^32^ Increasing (by 5%) the leak conductance (g_KL_) in TC_FO_ neurons (in order to mimic the increased tonic GABA‐A inhibition observed in genetic AS models^27^, ^28^) led to the appearance of SWDs at ~4 Hz (Figure 1A_1_,B_1_; Table S1), a frequency similar to that in humans with ASs.^2^, ^5^ Further increases in g_KL_ did not change the SWD frequency and duration though decreased and then prolonged the interictal period (Figure 1A_2_,A_3_; Table S1).

**FIGURE 2:**
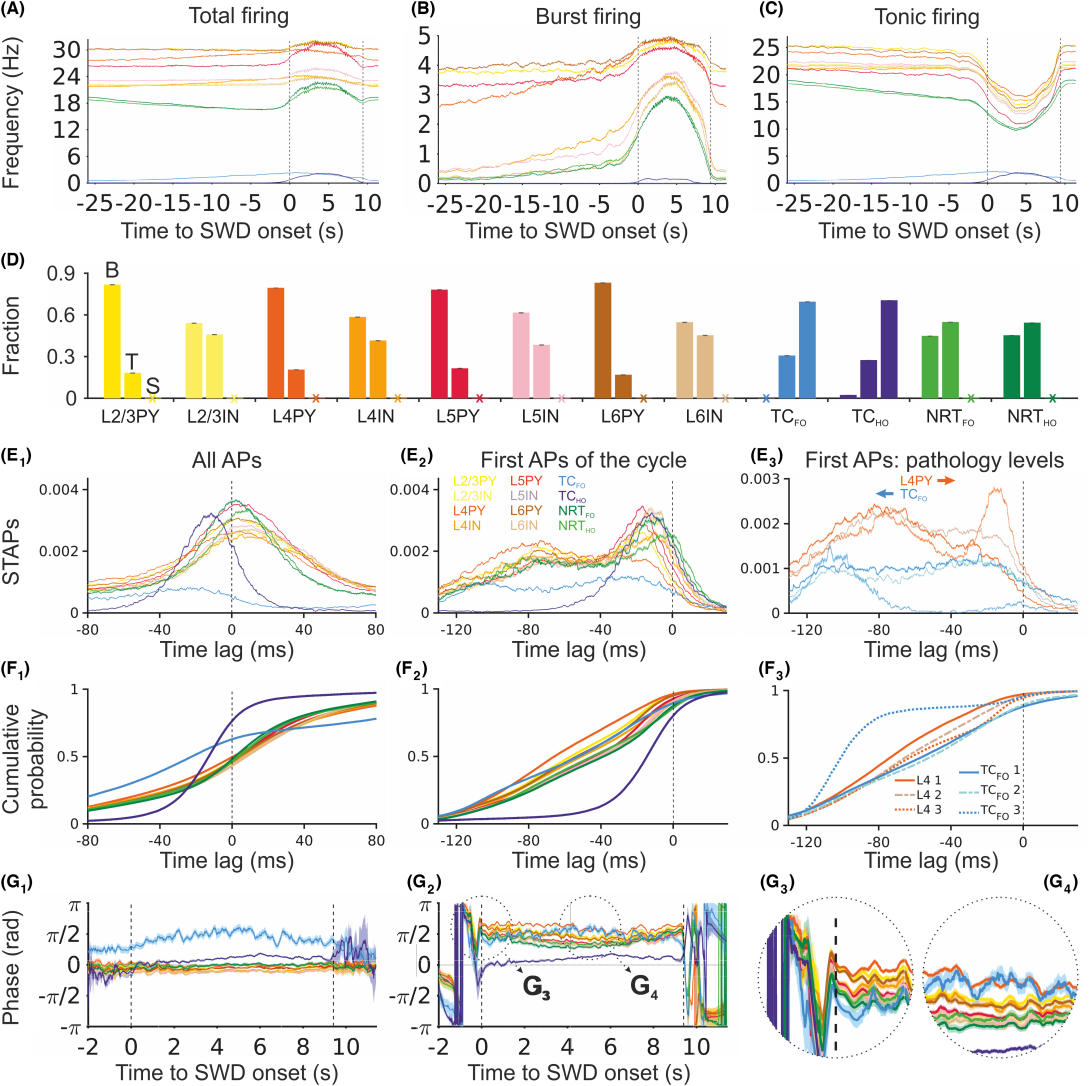
Firing properties during SWDs elicited by increased tonic GABA‐A inhibition of TC_FO_ neurons. (A–C) Interictal and ictal time evolution of total, burst, and tonic firing frequency for the indicated neuron types. Ictal and interictal periods were linearly scaled to their average durations. The shaded regions represent 95% confidence intervals. Dashed vertical black lines represent the onset and offset of the averaged SWD. Color‐code as in (E_2_). (D) Mean proportion of the indicated neurons showing burst and tonic firing (B and T column, respectively) and those that are silent (S column). Error bars indicate 95% confidence intervals. (E_1_) Cross‐correlations between all APs of different neuronal types and the SWD spike (SWD spike‐triggered action potentials: STAPs). Shaded regions are 95% confidence intervals. Dashed vertical line indicates the peak of the SWD spike. Color‐code as in (E_2_). (E_2_) Same as (E_1_) but only for the first AP in an SWD cycle. (E_3_) Same as (E_2_) but only for L4PY and TC_FO_ neurons for the three color‐coded g_KL_ values indicated in (F_3_) and Figure 1A_2_. Arrows indicate the shift of the firing peaks as g_KL_ is increased. (F_1‐3_) Cumulative AP probability corresponding to (E_1‐3_). (G_1_) Hilbert transform mean phase of APs of all cell types with respect to the SWD spike. Different SWDs were linearly scaled to the average duration SWD. Dashed vertical lines indicate the SWD onset and offset. Shaded regions represent 95% confidence intervals. Color code as in (E_2_). (G_2_) Same as (G_1_) but only for the first AP in an SWD cycle. (G_3,4_) Same as (G_2_) showing the enlarged regions circled in (G_2_). L2/3 PY, pyramidal neuron in cortical layers 2 and 3; L2/3 IN, interneuron in cortical layers 2 and 3; L4 PY, pyramidal neuron in cortical layer 4; L4 IN, interneuron in cortical layer 4; L5 PY, pyramidal neuron in cortical layer 5; L5 IN, interneuron in cortical layer 5; L6 PY, pyramidal neuron in cortical layer 6; L6 IN, interneuron in cortical layer 6; TC_FO_, first‐order TC neuron; TC_HO_, higher‐order TC neuron; NRT_FO_, first‐order NRT neuron; NRT_HO_, higher‐order NRT neuron.

At the single‐cell level, almost all neuronal populations increased their total firing during SWDs except TC_FO_ neurons which showed a decrease (Figures 1B_1_,B_2_ and 2A). The same was observed for ictal burst firing, whereas tonic, single action potential (AP) firing decreased (Figure 2B,C). Indeed, burst firing was the highest contributor to ictal activity in all excitatory and inhibitory cortical neurons (independent from their layer location), but was absent in TC_FO_ neurons and similar to tonic firing in all NRT neurons (Figure 2D). Notably, all cortical and NRT neurons were never silent during SWDs, whereas both TC_FO_ and TC_HO_ neurons were mostly silent ictally or fired tonically (Figure 2D). When considering the firing dynamics of all ictal APs, all neuronal populations fired at or just after the SWD spike except TC_FO_ and TC_HO_ neurons that fired ~30 and 20 ms, respectively, prior to the SWD spike (Figure 2E_1_,F_1_). This is also reflected in the firing phase evolution throughout the SWD with TC_FO_ and TC_HO_ cells showing a positive phase through most of the SWD (leading) while cortical cells showing zero or slightly negative phase over the same period (lagging) (Figure 2G_1_). However, when only the first AP of an SWD cycle was considered, all neurons fired ~10 ms before the SWD spike (Figure 2E_2_), and almost all neuron types had a smaller peak ~80 ms prior to the SWD spike (Figure 2F_2_). Notably, further increases in the tonic GABA‐A inhibition of TC_FO_ neurons moved the peak of the first AP in each cycle to the left and the right in TC_FO_ and layer 4 pyramidal (L4/PY) neurons, respectively (Figure 2E_3_,F_3_). Spike‐triggered action potential (STAP) histograms, however, do not decisively show which structures are leading during individual oscillation cycles. The temporal evolution of the phase of the first APs indicates that their phases do not remain stable (Figure 2G_2_). Indeed, whereas the cortex is leading during the initial few seconds of the SWD (Figure 2G_3_), the TC_FO_ cells briefly catch up and then gradually fall behind the cortical cells again (Figure 2G_2_,G_4_).

**FIGURE 3:**
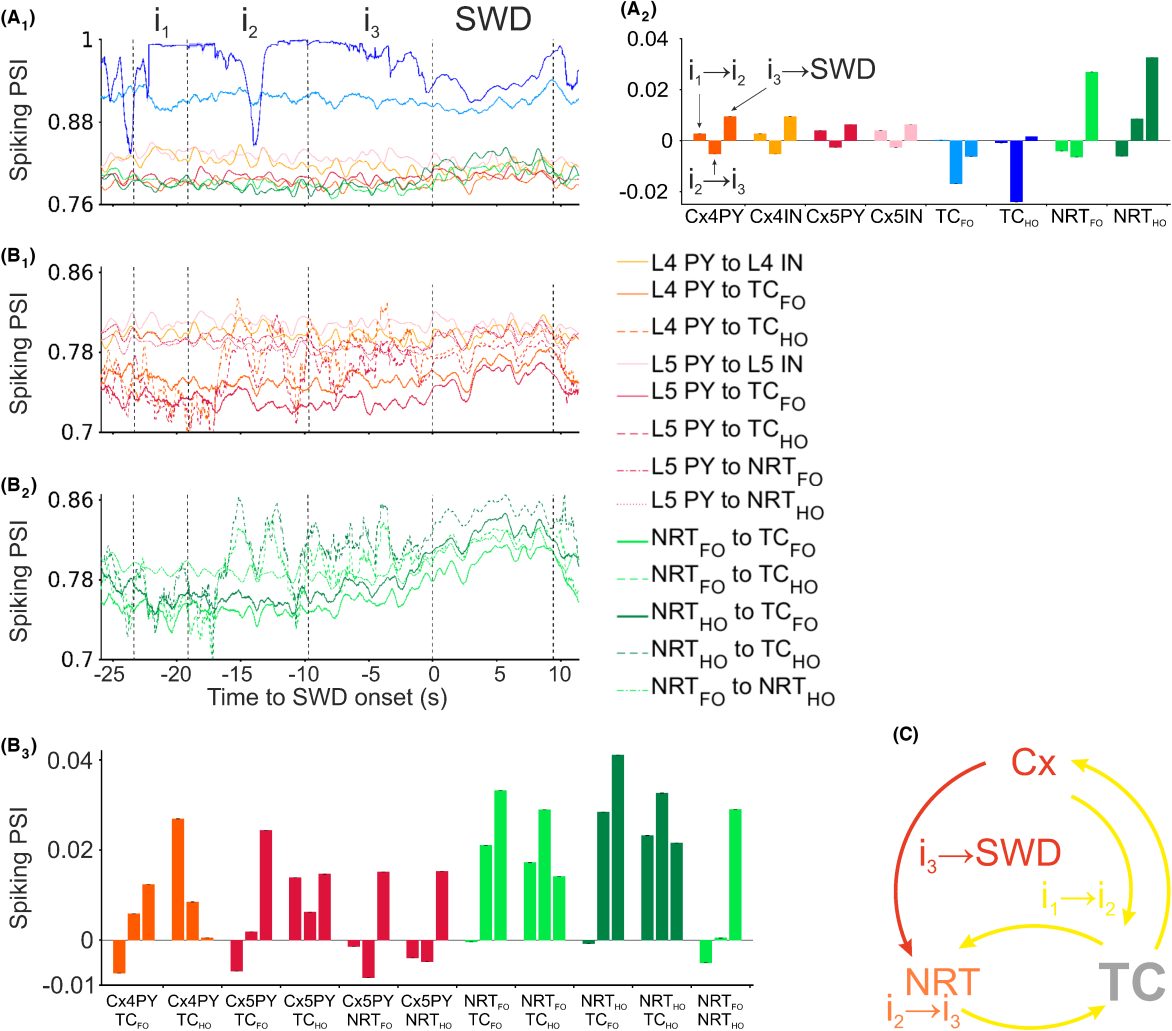
Time evolution of interictal and ictal firing synchrony for SWDs elicited by increased tonic GABA‐A inhibition of TC_FO_ neurons. (A_1_) Ictal and interictal mean phase synchronization index (PSI) of APs within a neuronal population (color‐code as in Figure 2E_2_). Ictal and interictal periods were linearly scaled to their average durations. Dashed vertical black lines indicate different parts of ictal and interictal periods: i_1_ marks a section from 1/6 to 1/3 of the interictal period, i_2_ marks the 1/3 to 2/3 section, i_3_ marks the final third of the interictal period, and the last two lines indicate the start and end of the ictal period. (A_2_) Changes in PSI during interictal and ictal periods. For each indicated neuronal population, the left bar is the PSI change between i_1_ and i_2_ (i_1_ → i_2_), the middle bar between i_2_ and i_3_ (i_2_ → i_3_), and the right bar between i_3_ and the ictal period (i_3_ → SWD). (B_1,2_) Evolution of ictal and interictal PSI between different cortical (B_1_) and thalamic (B_2_) populations (color‐code on the right). Vertical dashed black lines demarcate the same regions as in (A_1_). (B_3_) Changes in PSI of different neuronal populations over interictal and ictal periods. For each neuronal population pair, the three bars are as in (A_2_). (C) Schematic representation of the evolution of PSI. Brian areas and their connections shaded in yellow show increase in PSIs between i_1_ and i_2_ and represent the initial synchronization stage (corresponding to bar 1 in all comparison groups of A_2_ and B_3_). Orange and red colors mark PSI increases during the second synchronization stage (between i_2_ and i_3_) and the final synchronization stage (between i_3_ and the ictal period), respectively.

We then analyzed the temporal dynamics of firing synchrony within and between neuronal populations in the interictal and ictal periods (Figure 3). Within a given neural type, the stronger progressive increase in synchrony from interictal to ictal periods was observed in NRT_FO_ and NRT_HO_ neurons while the smallest increase occurred in TC_FO_ and TC_HO_ neurons (Figure 3A_1,2_). Among different populations, those involving all possible pairs of thalamic neurons showed the highest progressive synchrony as did the layer 5 pyramidal neurons (L5PY) pairs with either NRT or TC neurons, whereas the synchrony between L4PY and TC_HO_ neurons gradually decreased (Figure 3B_3_). Thus, in summary, the temporal dynamics of increased synchrony progress from thalamic and cortical neuron pairs to NRT neuron pairs and then to cortical and NRT neuron pairs (Figure 3C).

### 3.2 SWD generation by other selective alterations in inhibitory and excitatory conductances

**FIGURE 4:**
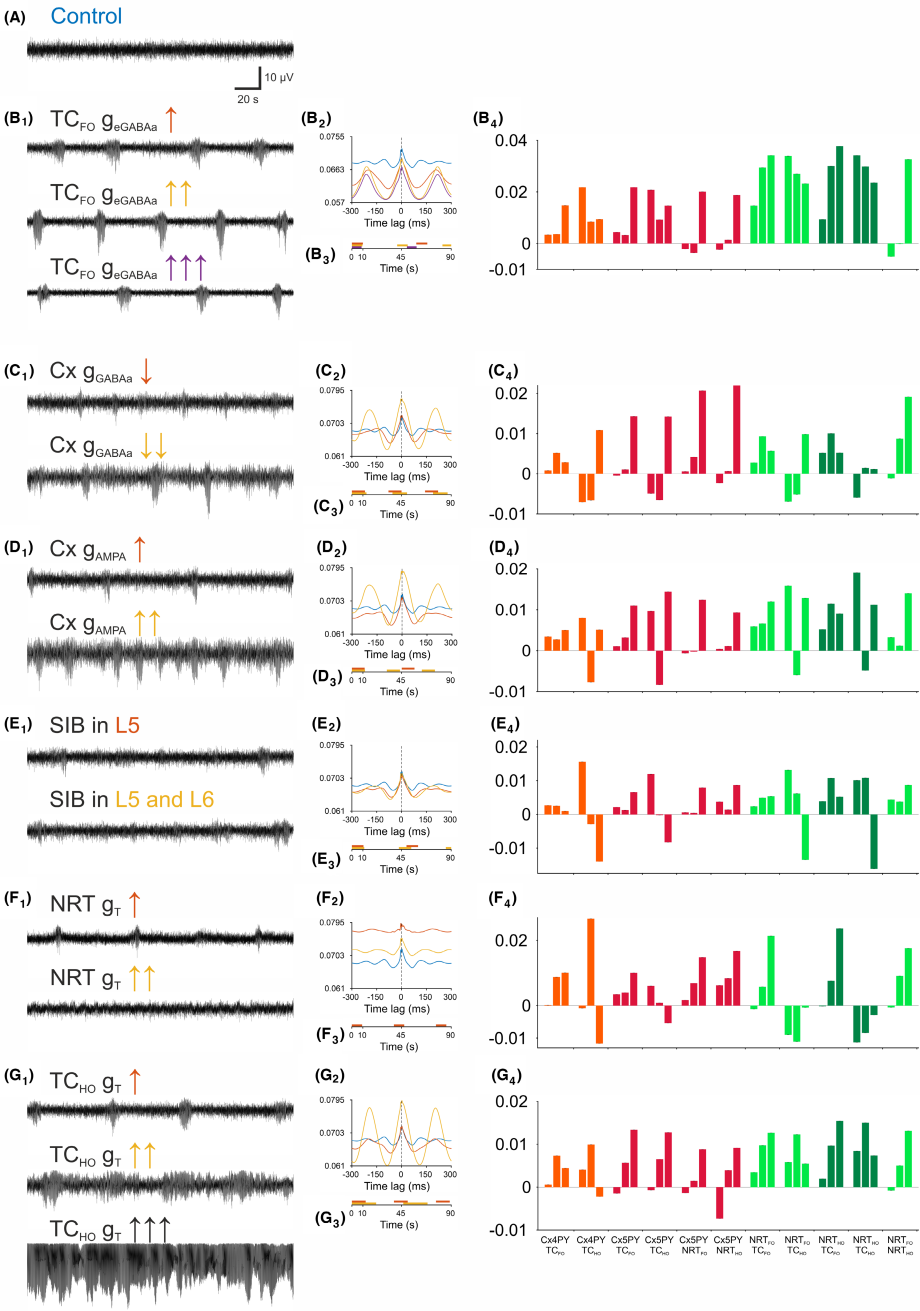
Thalamic and cortical abnormalities can independently induce SWDs. (A) EEG showing a period of simulated desynchronized state. (B_1_) SWDs elicited by progressive increases in the extrasynaptic GABA‐A conductance (g_eGABAa_) of TC_FO_ neurons. (B_2_) Cross‐correlations between APs of all cells and EEG (over a 20 min simulation period) with increased TC_FO_ neuron g_eGABAa_. Color code as in B1. Shaded regions represent 95% confidence intervals. (B_3_) Schematic SWD timeline showing SWD duration and frequency of occurrence for different TC_FO_ neuron g_eGABAa_ levels. (B_4_) Change in the firing PSI between the indicated neuronal populations in (G_4_). For each neuronal population pair, the left bar is the PSI change between i_1_ and i_2_ (i_1_ → i_2_), the middle bar between i_2_ and i_3_ (i_2_ → i_3_), and the right bar between i_3_ and the ictal period (i_3_ → SWD), as indicated in Figure 3A_2_. (C_1‐4_) same as (B_1‐4_) but showing SWDs following decreases in GABA‐A conductance (g_GABAa_) of all cortical neurons. (D_1‐4_) same as (B_1‐4_) but showing SWDs following increases in the cortical AMPA receptor conductance (g_AMPA_). (E_1‐4_) same as (B_1‐4_) but showing SWDs elicited by the addition of strongly intrinsically bursting (SIB) neurons in cortical layer 5 (L5) only or in both L5 and cortical layer 6 (L6). (F_1‐4_) same as (B_1‐4_) but showing SWDs after increases in the T‐type Ca^2+^ conductance (g_T_) of NRT cells. (G_1‐4_) same as (B_1‐4_) but showing SWDs after increases in the T‐type Ca^2+^ conductance (g_T_) of TC_HO_ cells. Note the absence status reached the highest increase of g_T_ in these thalamic neurons.

We first checked whether SWDs could be generated by an increase in conductance of the extrasynaptic GABA‐A receptors (g_eGABAa_) of TC_FO_ neurons instead of indirectly increasing the g_KL_ of these neurons. As shown in Figure 4B_1‐4_, progressive enhancement of this conductance reliably elicited SWDs. Moreover, selectively decreasing (by 10% and 25%) the conductance of the phasic GABA‐A inhibition (g_GABAa_) in all cortical neurons led to SWDs (as observed in vivo experiments^33^, ^34^, ^35^, ^36^) of progressively larger amplitude and increasingly longer interictal periods (Figure 4C_1‐3_; Table S2). Compared to the SWDs generated by an increase in thalamic tonic GABA‐A inhibition, stronger synchrony was observed between L5PY and all NRT neuron pairs as well as between L4PY and TC_HO_ neuron pairs in the simulated activity induced by a decreased cortical g_GABAa_ (Figure 4C_4_).

Progressively increasing (by 5% and 40%) the conductance of cortical AMPA receptors (g_AMPA_) also led to more frequent SWDs of increasing amplitude (Figure 4D_1,3_) (Table S2) and lower synchrony between L5PY and NRT neurons, compared to SWDs elicited by decreased cortical g_GABAa_ (Figure 4D_4_).

A higher number of cortical strongly intrinsically bursting (SIB) neurons have been reported in genetic rat models of ASs^37^, ^38^: its implementation in the model indeed generated SWDs although of a small amplitude compared to changes in other conductances (Figure 4E_1‐3_; Table S2) and with a characteristic interictal‐to‐ictal decrease in synchrony in L4PY‐TC_HO_, L5PY‐TC_HO_, NRT_HO_‐TC_FO_, and NRT_HO_‐TC_HO_ neuron pairs (Figure 4E_4_).

Finally, increasing the conductance of the T‐type Ca^2+^ channels (g_T_) in all NRT neurons, as it has been observed in both humans and experimental models of ASs,^39^, ^40^ led to brief, small‐amplitude SWDs, which, notably, were abolished by a further increase in this conductance (Figure 4F_1‐4_). Moreover increasing (by 5% and 10%) g_T_ in TC_HO_ neurons (Table S2) generated progressively longer SWDs, ultimately leading to absence status (Figure 4G_1,3_), and a gradual increase in synchrony among almost all neuronal pairs (Figure 4G_4_).

### 3.3 Critical conductances of simulated SWDs

**FIGURE 5:**
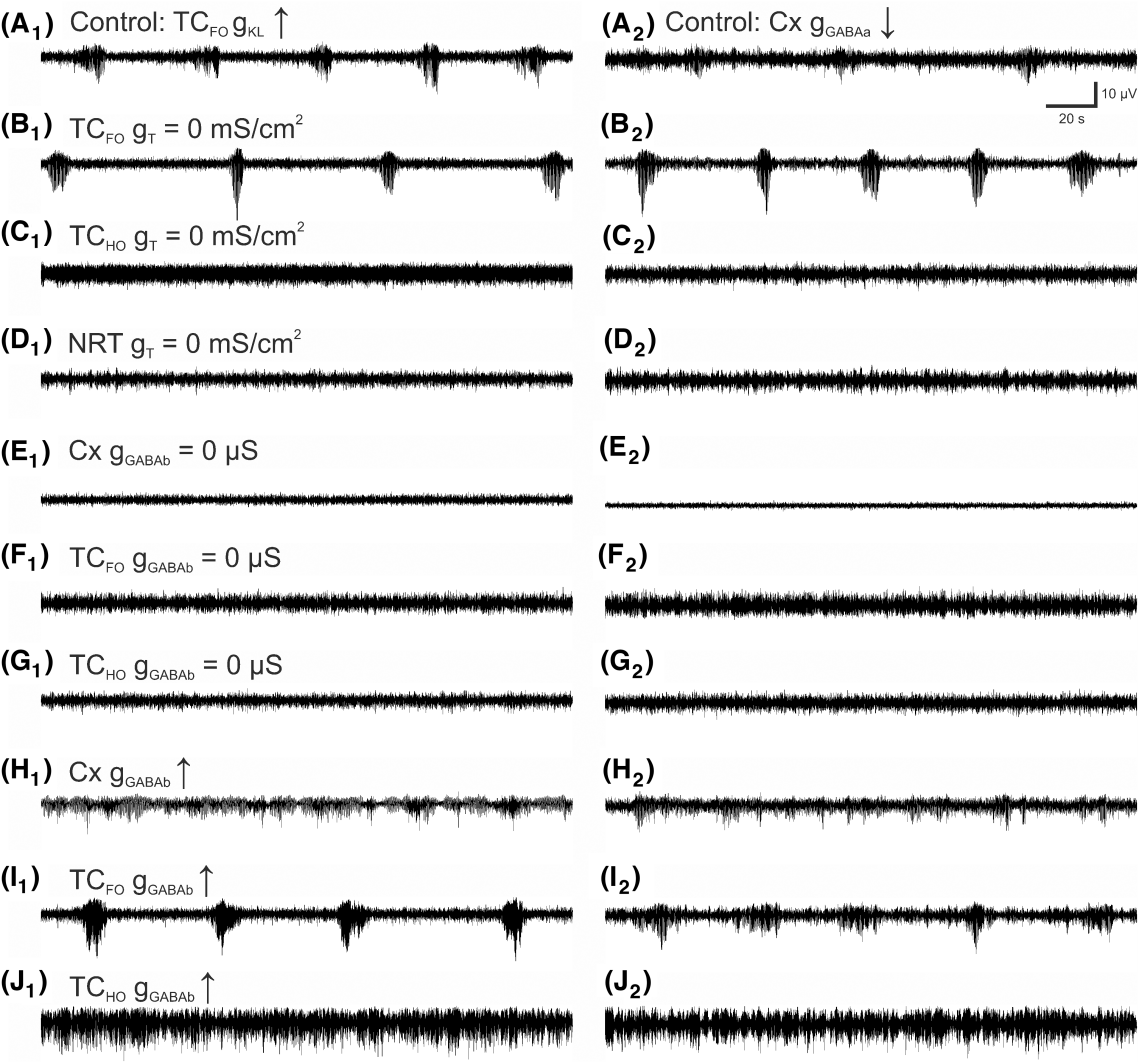
Essential contribution of various voltage‐ and transmitter‐gated conductances to simulated SWDs. (A_1_) Control SWDs elicited by increased g_KL_ in TC_FO_ neurons. (A_2_) Control SWDs elicited by decreased neocortical g_GABAa_. (B_1,2_) SWDs persist after blocking g_T_ in TC_FO_ neurons. (C_1,2_) SWDs are not generated when g_T_ is blocked in TC_HO_ neurons. (D_1,2_) SWDs are not elicited when g_T_ is blocked in all NRT neurons. (E_1,2_) SWDs are blocked after removing g_GABAb_ in all neocortical neurons. (F_1,2_) SWDs are blocked after removing g_GABAb_ in TC_FO_ neurons. (G_1,2_) SWDs are blocked after removing g_GABAb_ in TC_HO_ neurons. (H_1,2_) Smaller‐amplitude, almost continuous SWDs are elicited when g_GABAb_ is increased in neocortical neurons. (I_1,2_) the SWD amplitude and the interictal period are increased when g_GABAb_ is increased in TC_FO_ neurons. (J_1,2_) Absence status is generated when g_GABAb_ is increased in TC_HO_ neurons.

Having established that our model reproduces SWDs elicited by either increasing thalamic tonic or decreasing cortical phasic GABA‐A inhibition, we next studied which effect other conductances have on these simulated SWDs. Removing g_T_ from TC_FO_ neurons did not abolish SWDs, as recently reported,^41^ but actually increased their amplitude and decreased the interictal period duration (Figure 5B_1,2_). In contrast, removing g_T_ from TC_HO_ neurons abolished SWDs elicited by both increased thalamic tonic and decreased cortical phasic GABA‐A inhibition (Figure 5C_1,2_) as did g_T_ removal from all types of NRT neurons (Figure 5D_1,2_).

Blocking the conductances of GABA‐B receptors (g_GABAb_) in either all cortical or thalamic neurons abolished SWDs generated by increased thalamic tonic and decreased cortical phasic GABA‐A inhibition (Figure 5E_1,2_,F_1,2_,G_1,2_) as shown experimentally.^42^, ^43^, ^44^, ^45^ In contrast, increasing g_GABAb_ in cortical neurons decreased the amplitude of SWDs and markedly increased their duration (Figure 5H_1,2_). Enhancing g_GABAb_ in TC_FO_ neurons increased the amplitude and the interictal period of SWDs elicited by the increased thalamic tonic GABA‐A inhibition (Figure 5I_1_), whereas it decreased the interictal period of the SWDs simulated by a decreased cortical phasic GABA‐A inhibition (Figure 5I_2_). Finally, increasing g_GABAb_ in TC_HO_ neurons led to absence status in both models (Figure 5J_1,2_).

**FIGURE 6:**
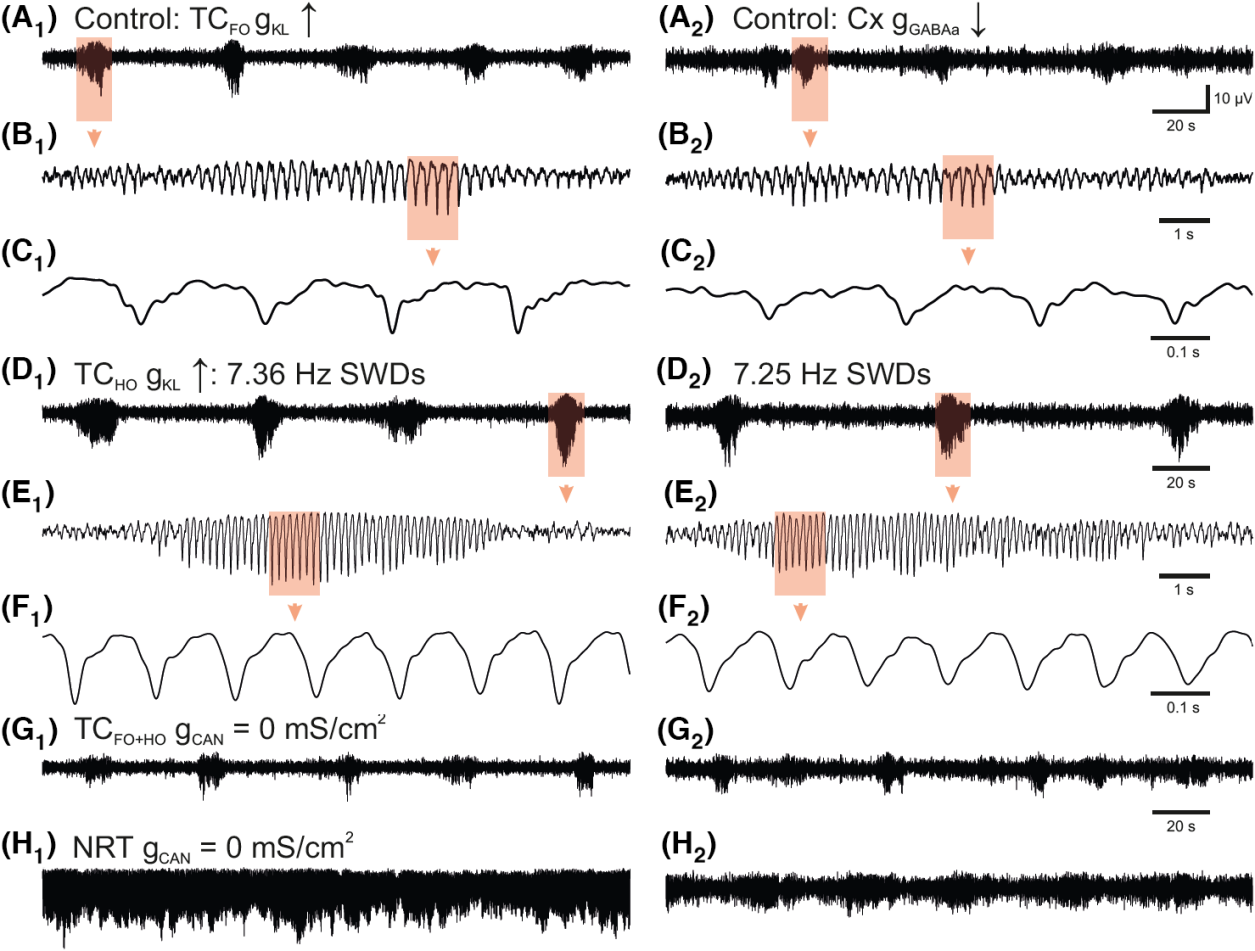
Effect of I_CAN_ and control of SWD frequency by g_KL_ of TC_HO_ neurons. (A_1_) Control SWDs elicited by increased g_KL_ in TC_FO_ neurons. (A_2_) Control SWDs elicited by decreased neocortical g_GABAa_. Highlighted areas are enlarged below the corresponding traces (B and C). (D_1,2_) Decreasing g_KL_ in TC_HO_ neurons leads to SWDs at ~7 Hz (typical of AS models) in both the model with increased g_KL_ of TC_FO_ neurons and that with decreased g_GABAa_ in cortical neurons. Highlighted areas are enlarged below the corresponding traces (E and F). (G_1,2_) Blocking g_CAN_ in TC_FO_ and TC_HO_ neurons has very little effect on the SWDs generated by both models. (H_1_) Blocking g_CAN_ in all NRT neurons transforms the well‐separated SWDs elicited by the increased g_KL_ in TC_FO_ neurons into absence status. (H_2_) Blocking g_CAN_ in all NRT neurons of the model with decreased g_GABAa_ in cortical neurons markedly prolongs the duration of SWDs.

Next, we investigated which conductance was critical for determining the simulated intra‐SWD frequency as it represents a major difference between ASs in human and animal models. We found that both in the models with an increased thalamic tonic and decreased cortical phasic GABA‐A inhibition, a reduction of the g_KL_ of TC_HO_ neurons increased the frequency of SWDs from ~4 Hz to the 7–8 Hz (compare Figure 6A_1,2_ with D_1,2_), a value that is typical of mouse and rat genetic and pharmacological models.^1^, ^3^, ^4^, ^5^

Finally, since the non‐selective cation conductance (g_CAN_) plays a key role in some EEG waves of natural^46^, ^47^, ^48^ and simulated^23^ sleep and its involvement in ASs has not been studied before, we investigated whether it is necessary for simulated SWDs. Removing g_CAN_ from all TC neurons had little effect on SWDs (Figure 6G_1,2_). In contrast, removal of (g_CAN_) from all NRT neurons led to absence status in the model with increased g_KL_ of TC_FO_ neurons (Figure 6H_1_) and markedly prolonged SWDs in the model with decreased g_GABAa_ in cortical neurons (Figure 6H_2_).

## 4 DISCUSSION

The main finding of this study is the ability to faithfully reproduce SWDs at the 4 Hz frequency observed in human ASs by single modifications of neocortical or thalamic conductances in a corticothalamic model that faithfully reproduces the intrinsic and network activity observed in neocortical and thalamic territories during natural sleep.^23^ To the best of our knowledge, this is the most detailed large‐scale model dedicated to simulating SWDs, and its component parts and their connectivity patterns were replicated with a high degree of fidelity to experimental data.^22^ Constructing a multipurpose model guards against an implementation bias of favoring a particular (patho)physiological regime. In fact, no previous attempt at modeling SWDs had this level of physiological validity.

### 4.1 Model limitations

Notwithstanding, our model has a number of limitations (see Dervinis and Crunelli^23^ for details). In the absence of direct measurements, the T‐type Ca^2+^ current implemented in various types of neocortical neurons was guided by the ability of these neurons to faithfully reproduce intrinsic slow (<1 Hz) waves.^23^ Moreover, although no detailed parameters exist for the persistent Na^+^ current in NRT neurons, this current (with biophysical properties similar to those reported for TC neurons^46^) had to be introduced in NRT neurons in order to faithfully reproduce the intrinsic slow (<1 Hz) waves observed in in vitro studies.^48^ Finally, since no voltage‐clamp study has been performed in higher‐order thalamic nuclei, the biophysical properties of the conductances of TC_HO_ neurons were inferred from current‐clamp data and/or adapted from TC_FO_ neurons.^49^, ^50^, ^51^, ^52^

### 4.2 Simulation strength

The solidity of our simulated SWDs is supported by two major findings. First, our model faithfully reproduces the three main EEG waves generated by corticothalamic networks during sleep, that is, spindle, delta, and slow (<1 Hz) waves,^23^ and these natural rhythms are only minimally affected by the different changes in single voltage‐ and transmitter‐gated conductances that lead to SWDs. Second, our model is capable of reproducing many experimental findings after implementing the different abnormalities that are known to be present in humans with, and genetic models of, ASs.

In particular, our model generates ~4 Hz SWDs following:

1. blockade of neocortical phasic GABA‐A inhibition, as shown experimentally following intracortical injection of the weak and potent GABA‐A antagonists penicillin and bicuculline, respectively^33^, ^34^, ^35^, ^36^, ^53^, ^54^, ^55^;
2. increase in the tonic GABA‐A inhibition of TC_FO_ neurons, by directly increasing the function of extrasynaptic GABA‐A receptors or indirectly increasing the g_KL_ of these neurons, as shown in different genetic models of ASs,^27^ that is, the GAERS (Genetic Absence Epilepsy Rats from Strasbourg) rats and the stargazer and lethargic mouse models;
3. enhancement of GABA‐B inhibition in either thalamic or cortical territory, as shown by the generation and aggravation of SWDs in normal mice and rats and genetic AS models, respectively, following systemic, intracortical, and intrathalamic injection of GABA‐B receptor agonists^42^, ^43^, ^44^, ^45^;
4. increase in the T‐type Ca^2+^ channel function in TC_HO_ neurons, as reported by Gorji et al.^49^ and Seidenbecher et al.^50^;
5. increase in the T‐type Ca^2+^ channel function in NRT neurons, as reported by Chen et al.^39^ and Cain et al.^40^; and
6. enhancement of the number of intrinsically bursting cells in layers 5/6, as observed in the Wistar Albino Glaxo Rats from Rijswijk^37^ and the GAERS genetic models of ASs.^38^

Our simulations also show that SWDs are abolished or reduced following (1) blockade of cortical or thalamic GABA‐B receptors as observed in different genetic and pharmacological models of ASs following systemic, intracortical, or intrathalamic injection of selective GABA‐B receptor antagonists^40^, ^41^, ^42^, ^43^; and (2) removal of T‐type Ca^2+^ channels in NRT neurons, as seen following intra‐NRT infusion of TTA‐P2,^39^ a potent and selective blocker of these channels,^56^ in GAERS rats. In contrast, simulated SWDs are unaffected by blocking T‐type Ca^2+^ channels in TC_FO_ neurons as reported by McCafferty et al.^41^ Notably, an increase in g_T_ of all NRT neurons, as observed in humans and models of ASs,^39^, ^40^ only led to brief, small‐amplitude SWDs, clearly indicating that this thalamic abnormality is not capable alone to induce a solid absence phenotype.

Finally, the strength of our results is also supported by their similarities with the following experimental findings:

1. TC_FO_ neurons, as those in the ventrobasal thalamus, are mostly silent during SWDs^41^, ^57^;
2. burst firing of NRT and cortical neurons increases during SWDs^41^, ^58^;
3. tonic firing is reduced in all types of cells, as shown experimentally,^41^ except in TC_HO_ cells for which no data are available at present;
4. the burst firing of cortical pyramidal neurons and interneurons in all layers is increased ictally compared to interical periods^38^, ^41^, ^59^;
5. the transition between sleep and quiet wakefulness is the vigilance state where most SWDs occur.^60^, ^61^

### 4.3 Predictions from simulated SWDs

A number of key testable predictions originate from the results of our study:

1. T‐type Ca^2+^ channel‐mediated burst firing in TC_HO_ neurons is necessary to elicit SWDs;
2. depolarization of TC_FO_ neurons prevents SWD generation or interferes with ongoing SWDs;
3. I_CAN_ of NRT neurons is essential for termination of SWDs;
4. g_KL_ of TC_HO_ neurons is a key determinant of SWD frequency;
5. absence status occurs when (i) blocking I_CAN_ in NRT neurons, (ii) strongly increasing g_T_ in TC_HO_ neurons, and (iii) increasing g_GABAb_ in TC_HO_ neurons. These results provide the first mechanistic insight into absence status and have a strong translational significance since both T‐type Ca^2+^ channel blockers and GABA‐B receptor antagonists are being trialed in human cohorts.^5^

## CONFLICT OF INTEREST STATEMENT

The authors declare no conflict of interest.

## Supporting information


Appendix S1

## Data Availability

The model codes are available to download from Github via Zenodo (https://doi.org/10.5281/zenodo.7724411 and https://doi.org/10.5281/zenodo.7724443).
