# Supplementary material for: Spike‐and‐wave discharges of absence seizures in a sleep waves‐constrained corticothalamic model
Source: CNS Neurosci Ther. 2023 Apr 10;30(3):e14204. doi: 10.1111/cns.14204 (PMC10915988; doi:10.1111/cns.14204)
Supplement: Supplementary file 1 — Appendix S1 [file CNS-30-e14204-s001.pdf]

Spike-and-wave discharges of absence seizures in a sleep waves-constrained corticothalamic model

Martynas Dervinis and Vincenzo Crunelli

Neuroscience Division, School of Bioscience, Cardiff University, Cardiff CF10 3AX, UK

SUPPLEMENTARY INFORMATION

Supplementary Figure 1 page 2

Supplementary Figure 2 page 4

Supplementary Table 1 page 6

Supplementary Table 2 page 7

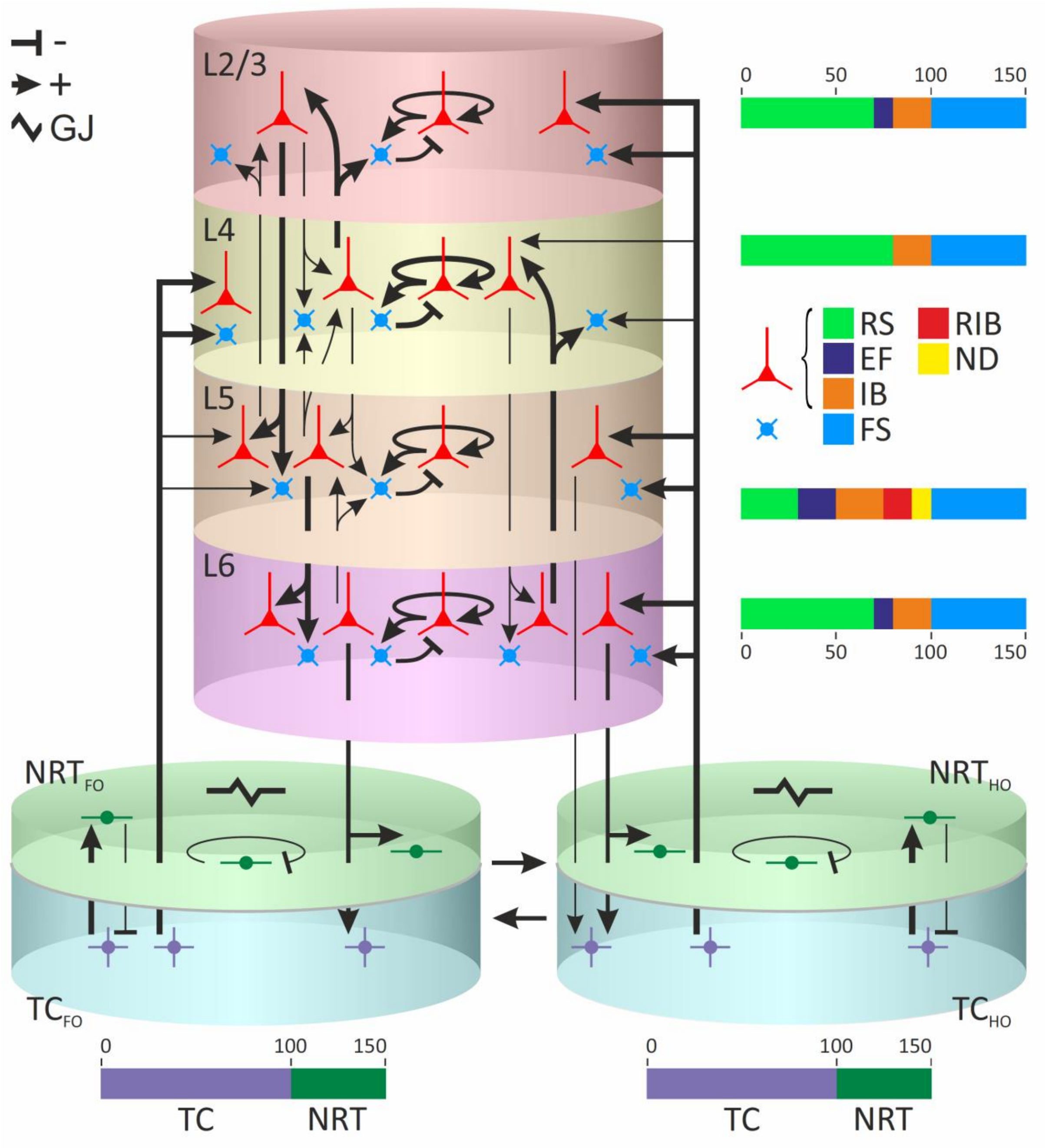

Figure S1. Corticothalamic model architecture.

The corticothalamic model consists of 900 neurons distributed in distinctly coloured layers of a single cortical column and two coloured-coded thalamic sectors, a first- and a higher-order sector. Each cortical layer contains of 100 excitatory neurons (detailed neuronal populations are in the colour-coded bars on the right) and 50 fast-spiking (FS) inhibitory neurons. Thalamic cylinders (of both first-order and higher-order sectors) contain 100 thalamocortical (TC) neurons and 50 nucleus reticularis thalami (NRT) neurons. Sharp and blunt arrows represent excitatory and inhibitory synaptic connections, respectively, with the line thickness indicating

the synaptic connection strength (see actual values in Supplementary Table 1). The lightning symbol indicates gap junctions that are present only between NRT neurons.

L2/3: cortical layer 2/3

L4: cortical layer 4

L5: cortical layer 5

L6: cortical layer 6

RS: regular spiking neuron

EF: early firing neuron

IB: intrinsically bursting neuron

RIB: repetitive intrinsically bursting neuron

ND: network driver neuron

FS: fast spiking neuron

TC_{FO}: thalamocortical neurons of first-order thalamic nucleus

TC_{HO}: thalamocortical neurons of higher-order thalamic nucleus

NRT_{FO}: nucleus reticularis thalami neurons connected to first-order nucleus,

NRT_{HO}: nucleus reticularis thalami neurons connected to higher-order nucleus.

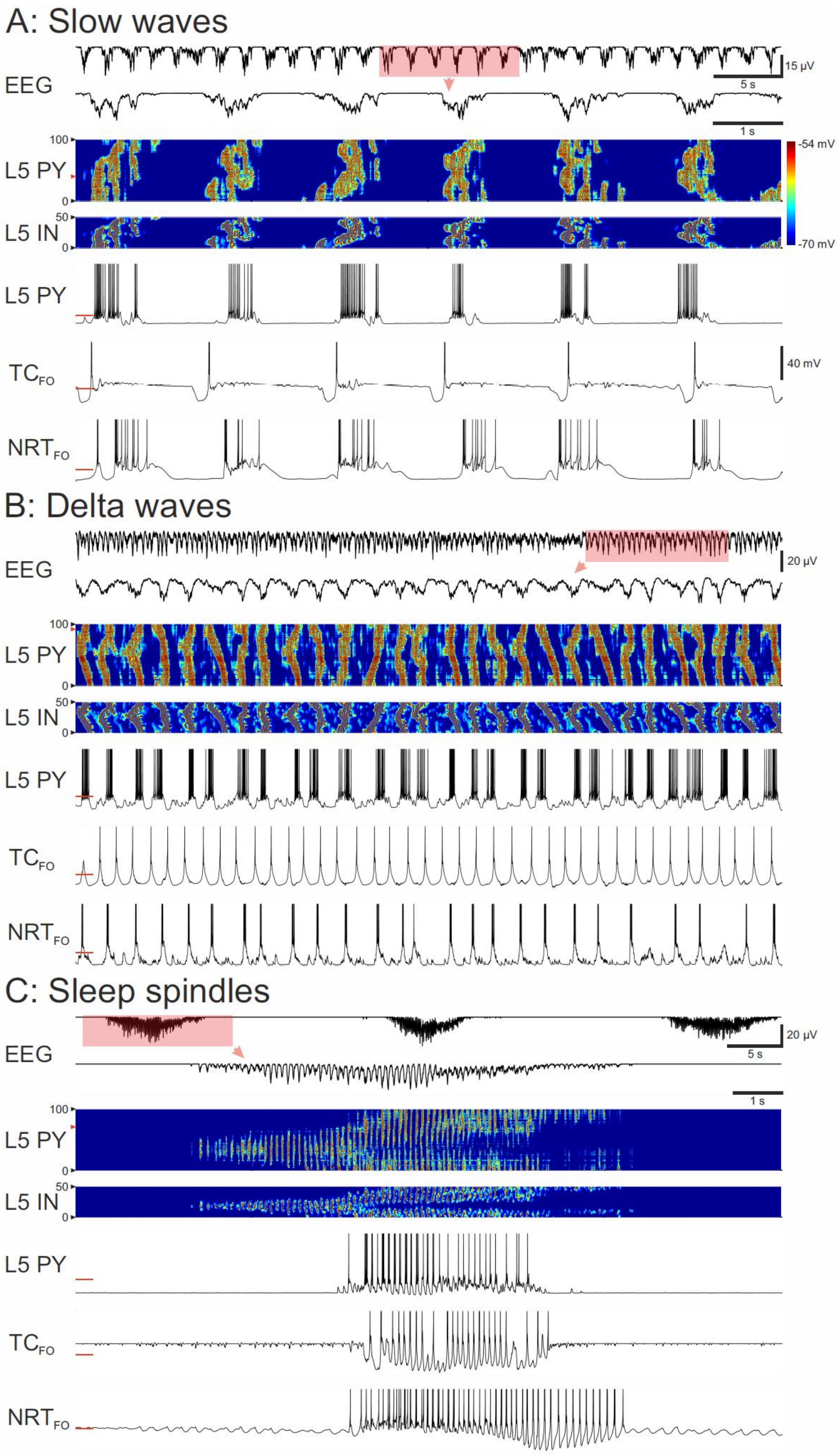
A: Slow waves
EEG
15 µV
5 s
1 s
L5 PY
L5 IN
-54 mV
-70 mV
L5 PY
TC_FO
40 mV
NRT_FO
B: Delta waves
EEG
20 µV
L5 PY
L5 IN
L5 PY
TC_FO
NRT_FO
C: Sleep spindles
EEG
20 µV
5 s
1 s
L5 PY
L5 IN
L5 PY
TC_FO
NRT_FO

Figure S2. Simulated physiological sleep waves in the corticothalamic model.

A, Simulated slow (<1 Hz) waves (EEG) and corresponding colour-coded membrane potential graphs (middle graphs) and membrane potential waveform (bottom traces) for the indicated neuronal populations. B, Simulated delta waves (EEG) and corresponding colour-coded membrane potential graphs (middle graphs) and membrane potential waveform (bottom traces) for the indicated neuronal populations. C, simulated sleep spindles (EEG) and corresponding colour-coded membrane potential graphs (middle graphs) and membrane potential waveform (bottom traces) for the indicated neuronal populations. In A, B and C, i) highlighted EEG sections are enlarged in the graphs and traces below; ii) red arrow in the colour-code membrane potential graphs indicate the neuron whose membrane potential trace is depicted below; and iii) red line in the membrane potential traces indicates -60 mV. L5 PY: layer 5 cortical pyramidal neuron; L5 IN: layer 5 cortical interneuron; TC_{FO}: thalamocortical neuron of first-order thalamic nucleus; NRT_{FO}: nucleus reticularis thalami neuron connected to first-order thalamic nucleus.

Table S1. Parameters of SWDs induced by pathologies with tonic inhibition of TC_{FO} cells.

Pathology type	Inhibition by I_{KL}			Inhibition by extra-synaptic I_{GABAa}		
Pathology level	1	2	3	1	2	3
SWD incidence (min^{-1})	1.16	1.57	1.01	1.01	1.47	1.16
Mean SWD duration (s)	8.76 ± 0.29	9.43 ± 0.19	9.32 ± 0.25	9.97 ± 0.31	9.52 ± 0.17	8.76 ± 0.17
Mean interictal period (s)	42.08 ± 1.35	27.93 ± 0.71	47.71 ± 1.68	48.39 ± 3.59	31.51 ± 0.67	41.1 ± 0.81
Time spent in SWDs (%)	17.23	25.23	16.34	17.09	23.2	17.57
Mean spike-to-wave amplitude (μV)	5.83 ± 0.0057	7.12 ± 0.005	6.78 ± 0.007	6.45 ± 0.006	8.1 ± 0.006	7.93 ± 0.0076
Mean PSI	0.64 ± 0.00002	0.7 ± 0.00001	0.69 ± 0.00002	0.67 ± 0.00002	0.72 ± 0.00001	0.73 ± 0.00002
AP cross-correlation amplitude	0.02	0.026	0.019	0.024	0.03	0.022
AP-EEG correlation amplitude	0.0078	0.0098	0.0071	0.0085	0.0114	0.0091
Intra-SWD freq. (Hz)	4.79	4.75	4.66	4.84	4.65	4.65
Slowing down of intra-SWD freq. (yes/stable/increase)	Yes	Yes	Increase	Yes	Yes	Increase
Presence of poly-spikes (yes/no)	Yes	Yes	Yes	Yes	Yes	Yes
Leading structure	L4	L4	L4	L4	L4	L4
Mean θ of 1st L2/3 AP in 1st sec of SWD (rad)	1.98 ± 0.052	1.94 ± 0.05	1.97 ± 0.057	2.04 ± 0.059	1.85 ± 0.055	2.08 ± 0.064
Mean θ of 1st L4 AP in 1st sec of SWD (rad)	2.13 ± 0.041	2.045 ± 0.042	2.01 ± 0.054	2.25 ± 0.04	2.02 ± 0.046	2.16 ± 0.058
Mean θ of 1st L5 AP in 1st sec of SWD (rad)	1.69 ± 0.062	1.61 ± 0.059	1.66 ± 0.063	1.73 ± 0.071	1.52 ± 0.063	1.73 ± 0.074
Mean θ of 1st L6 AP in 1st sec of SWD (rad)	1.91 ± 0.055	1.87 ± 0.053	1.95 ± 0.059	1.98 ± 0.063	1.83 ± 0.059	2.02 ± 0.068
Mean θ of 1st NRT_{FO} AP in 1st sec of SWD (rad)	1.59 ± 0.09	1.51 ± 0.082	1.31 ± 0.11	1.7 ± 0.096	1.5 ± 0.092	1.5 ± 0.12
Mean θ of 1st NRT_{HO} AP in 1st sec of SWD (rad)	1.41 ± 0.108	1.37 ± 0.093	1.31 ± 0.099	1.47 ± 0.12	1.32 ± 0.102	1.38 ± 0.11
Mean θ of 1st TC_{FO} AP in 1st sec of SWD (rad)	1.51 ± 0.134	1.25 ± 0.15	1.35 ± 0.33	1.53 ± 0.15	1.23 ± 0.16	1.45 ± 0.41
Mean θ of 1st TC_{HO} AP in 1st sec of SWD (rad)	-0.23 ± 0.137	-0.017 ± 0.104	0.4 ± 0.072	0.18 ± 0.09	-0.0085 ± 0.085	0.38 ± 0.065
Fraction of bursting in TC_{FO} cells during SWDs (%)	0 ± 0	0 ± 0	0.0034 ± 0.00039	0 ± 0	0 ± 0	0 ± 0
Fraction of tonic spiking in TC_{FO} cells during SWDs (%)	42.87 ± 0.26	30.62 ± 0.22	5.43 ± 0.038	38.49 ± 0.0024	24.93 ± 0.0017	8.94 ± 0.05

Abbreviations: PSI, phase synchronisation index; freq, frequency; θ, phase.

Table S2. Parameters of SWDs induced by pathologies other than tonic inhibition of TC_{FO} cells.

Pathology type	Reduced cortical $GABA_A$ conductance		Increased cortical AMPA conductance		SIB in the cortex		Increased I_T in NRT	Increased I_T in TC_{HO}	
Pathology level	1	2	1	2	SIB in L5	SIB in L5/6	1	1	2
SWD incidence (min^{-1})	1.82	1.62	1.32	1.85	1.16	1.37	1.57	1.57	1.32
Mean SWD duration (s)	11.84 ± 0.43	13.19 ± 0.41	11.52 ± 0.46	11.85 ± 0.23	10.49 ± 0.62	11.21 ± 0.4	9.34 ± 0.22	12.47 ± 0.39	21.96 ± 0.77
Mean interictal period (s)	21.3 ± 0.75	23.58 ± 0.84	33.62 ± 1.71	19.81 ± 0.8	39.01 ± 2.3	31.38 ± 1.33	28.76 ± 0.93	25.64 ± 1.01	24.62 ± 1.35
Time spent in SWDs (%)	35.72	35.87	25.53	37.44	21.19	26.32	24.52	32.72	47.15
Mean spike-to-wave amplitude (µV)	4.44 ± 0.002	6.85 ± 0.003	4.72 ± 0.003	8.24 ± 0.004	3.86 ± 0.004	4.25 ± 0.003	3.02 ± 0.004	4.93 ± 0.003	8.45 ± 0.002
Mean PSI	0.64 ± 0.00001	0.52 ± 0.000009	0.57 ± 0.00001	0.65 ± 0.00001	0.56 ± 0.00002	0.59 ±0.00001	0.54 ±0.00001	0.58 ± 0.00001	0.82 ± 0.00001
AP cross-correlation amplitude	0.019	0.043	0.019	0.051	0.015	0.018	0.0067	0.019	0.059
AP-EEG correlation amplitude	0.0077	0.014	0.0078	0.015	0.0062	0.0073	0.0025	0.0075	0.018
Intra-SWD freq. (Hz)	4.79	4.6	4.86	4.64	5.06	5.19	5.45	5.09	4.99
Slowing down of intra-SWD freq. (yes/stable/increase)	Yes	Increase	Yes	Increase	Stable	Increase	Increase	Stable	Stable
Presence of poly-spikes (yes/no)	Yes	Yes	Yes	Yes	Yes	Yes	Yes	Yes	Yes
Leading structure	L4	L4	L4	L4	L4	L4	L4	L4	L4
Mean θ of 1st L2/3 AP in 1st sec of SWD (rad)	1.77 ± 0.05	2.37 ± 0.19	1.91 ± 0.05	1.29 ± 0.05	1.98 ± 0.054	1.9 ± 0.051	2.24 ± 0.036	1.97 ± 0.045	1.55 ± 0.066
Mean θ of 1st L4 AP in 1st sec of SWD (rad)	2.29 ± 0.03	2.76 ± 0.11	2.17 ± 0.046	2 ± 0.035	2.19 ± 0.044	2.15 ± 0.041	2.68 ± 0.027	2.18 ± 0.037	2.02 ± 0.043
Mean θ of 1st L5 AP in 1st sec of SWD (rad)	1.45 ± 0.059	2.06 ± 0.26	1.66 ± 0.065	1.04 ± 0.051	1.72 ± 0.066	1.56 ± 0.064	2.08 ± 0.043	1.74 ± 0.053	1.13 ± 0.078

Mean θ of 1st L6 AP in 1st sec of SWD (rad)	1.7 ± 0.055	2.3 ± 0.22	1.86 ± 0.06	1.16 ± 0.05	1.93 ± 0.06	1.81 ± 0.059	2.25 ± 0.035	1.91 ± 0.048	1.42 ± 0.075
Mean θ of 1st NRT_{FO} AP in 1st sec of SWD (rad)	1.92 ± 0.055	2.4 ± 0.31	1.7 ± 0.09	1.69 ± 0.059	1.72 ± 0.088	1.71 ± 0.079	1.7 ± 0.098	1.75 ± 0.072	1.56 ± 0.088
Mean θ of 1st NRT_{HO} AP in 1st sec of SWD (rad)	1.69 ± 0.072	1.95 ± 0.47	1.42 ± 0.11	1.45 ± 0.073	1.4 ± 0.12	1.42 ± 0.11	0.59 ± 0.15	1.48 ± 0.096	1.05 ± 0.11
Mean θ of 1st TC_{FO} AP in 1st sec of SWD (rad)	1.88 ± 0.075	2.35 ± 0.39	1.7 ± 0.13	1.76 ± 0.07	1.68 ± 0.11	1.59 ± 0.1	2.44 ± 0.035	1.74 ± 0.094	1.87 ± 0.054
Mean θ of 1st TC_{HO} AP in 1st sec of SWD (rad)	0.23 ± 0.13	-0.09 ± 0.33	-0.19 ± 0.16	0.15 ± 0.12	-0.45 ± 0.16	-0.41 ± 0.16	-0.29 ± 0.14	-0.34 ± 0.13	-0.054 ± 0.089
Fraction of bursting in TC_{FO} cells during SWDs (%)	0 ± 0	0 ± 0	0 ± 0	0 ± 0	0 ± 0	0 ± 0	0.02 ±0.000007	0 ± 0	0 ± 0
Fraction of tonic spiking in TC_{FO} cells during SWDs (%)	63.4 ± 0.25	54.16 ± 0.31	60.03 ± 0.25	61.12 ± 0.26	61.02 ± 0.25	58.01 ± 0.26	88.16 ± 0.001	57.24 ± 0.26	56.9 ± 0.26

Abbreviations: PSI, phase synchronisation index; freq, frequency; θ, phase.
